# Structure-Function Analysis of the Transmembrane Protein AmpG from *Pseudomonas aeruginosa*

**DOI:** 10.1371/journal.pone.0168060

**Published:** 2016-12-13

**Authors:** Peizhen Li, Jun Ying, Guangjian Yang, Aifang Li, Jian Wang, Junwan Lu, Junrong Wang, Teng Xu, Huiguang Yi, Kewei Li, Shouguang Jin, Qiyu Bao, Kaibo Zhang

**Affiliations:** 1 School of Laboratory Medicine and Life Science/Institute of Biomedical Informatics, Wenzhou Medical University, Wenzhou, China; 2 School of Medicine, Lishui College, Lishui, China; 3 Wenling Women’s & Children’s Hospital, Wenling, China; 4 Department of Molecular Genetics and Microbiology, University of Florida, Gainesville, Florida, United States of America; Russian Academy of Medical Sciences, RUSSIAN FEDERATION

## Abstract

AmpG is a transmembrane protein with permease activity that transports meuropeptide from the periplasm to the cytoplasm, which is essential for the induction of the *ampC* encoding β-lactamase. To obtain new insights into the relationship between AmpG structure and function, comparative genomics analysis, secondary and tertiary structure modeling, site-directed mutational analyses and genetic complementation experiments were performed in this study. AmpGs from different genera of bacteria (*Escherichia coli*, *Vibrio cholerae* and *Acinetobacter baumannii*) could complement AmpG function in *Pseudomonas aeruginosa*. The minimal inhibitory concentration (MIC) to ampicillin is 512 μg/ml for wild type strain PAO1, while it is 32 μg/ml for an *ampG* deletion mutant strain (*PAO1*Δ*ampG*) with a corresponding decrease in the activity of the *ampC*-encoded β-lactamase. Site-directed mutagenesis of conserved AmpG residues (G29, A129, Q131 and A197) resulted in a loss of function, resulting in a loss of resistance to ampicillin in *PAO1*Δ*ampG*. The G29A, G29V, A129T, A129V, A129D, A197S and A197D mutants had lower resistance to ampicillin and significantly decreased activity of the AmpC β-lactamase. The G29A, G29V, A129V, A197S and A197D mutants had decreased *ampG* mRNA transcript levels. The A129T and A129D mutants had normal *ampG* mRNA transcript levels, but the function of the protein was drastically reduced. Our experimental results demonstrate that the conserved amino acids played essential roles in maintaining the function of AmpG. Combined with the AmpG structural information, these critical amino acids can be targeted for the development of new anti-bacterial agents.

## Introduction

With the widespread use of antibiotics, bacterial resistance has become a major problem for global public health. Resistance to anti-bacterial agents is progressing at a rapid rate, while the speed of new anti-bacterial agent development is relatively slow, resulting in fewer and fewer alternative anti-microbial agents for use in clinical settings [1]. β-lactam anti-bacterial agents, including penicillin and cephalosporins, are the most widely used antibiotics in clinic, but with the rapid emergence of resistance, treatment failure and recurrent infections have become a serious threat to human health [2]. It has become imperative to develop more effective countermeasures to control antibiotic resistance in clinical settings.

Bacterial resistance to β-lactam anti-bacterial agents is mainly due to the production of β-lactamases by bacteria. The AmpC type β-lactamase belongs to a class of serine cephalosporinases produced by certain gram-negative bacteria and cannot be inhibited by clavulanic acid [3]. This type of β-lactamase has a broader substrate spectrum than extended spectrum β-lactamases (ESBLs), which can be extended to all types of β-lactam anti-bacterial agents except carbapenems [1]. AmpC type β-lactamases, but not ESBLs can hydrolyze cefoxitin. There are several genes involved in the regulation of *ampC* expression, including *ampD*, *ampR*, *ampG* and *ampE* [4, 5]. The gene *ampG* encodes an inner membrane permease for the transportation of muropeptides through the cell membrane. The muropeptides are peptidoglycan catabolites that, upon entry into the cytoplasm, bind to AmpR to activate its transcriptional activator function and induce the production of AmpC type β-lactamase [6–8]. Kong KF et al. reported that *P*. *aeruginosa* appeared to have two *ampG* paralogs, *ampG* (PA4393) and *ampP* (PA4218) [9]. In a later study by Zhang et al. [10], PA4393 was demonstrated to have AmpG permease function, while PA4218 encoded a protein that does not have permease activity. In *P*. *aeruginosa* strains PAO1 and PAK, inactivation of the *ampG* genes drastically repressed the intrinsic β-lactam resistance, while *ampGh1* deletion had no effect on the resistance. Inactivation of *nagZ* or *ampG* fully restored the susceptibility and basal *ampC* expression in *ampD* or *dacB* mutants, but only *ampG* inactivation fully blocked *ampC* induction, resulting in reduction in the MIC of the potent AmpC inducer imipenem from 2 to 0.38 μg/ml [11]. Overall, *ampG* acts as a “gatekeeper” and plays an important role in the expression of the AmpC type β-lactamase.

The use of AmpG inhibitors together with β-lactam anti-bacterial agents would undoubtedly restore bacterial susceptibility and extend the usefulness of common β-lactams in clinic. Greater knowledge on the structure of the target protein will likely lead to useful information for development of an effective inhibitor. In this work, we investigated several conserved residues based on the predicted structure of the AmpG protein to locate potentially functional domains. This work can aid the design and synthesis of chemicals that can be utilized as inhibitors targeting the transport function of the AmpG protein. This approach may open new avenues for the development of antibacterial agents to treat infectious diseases.

## Materials and Methods

### Bacterial strains and plasmids

The strains and plasmids used or constructed in this work are listed in Table 1. *Pseudomonas aeruginosa* PAO1 (*P*. *aeruginosa* PAO1) and plasmid pUCP24 [10] were obtained from the Laboratory of Microbial Genetics, University of Florida, Gainesville, USA. *Escherichia coli* 7 (*E*. *coli* 7), *Vibrio cholerae* 03 (*V*. *cholerae* 03) and *Acinetobacter baumannii* 2089 (*A*. *baumannii* 2089) are wild strains isolated from the First Affiliated Hospital of Wenzhou Medial University, China.

**Table 1:** Bacterial strains and plasmids used in this work.

Strain or plasmid	Relevant characteristic (s)	Reference/source
**Strains**		
*E*. *coli* DH5α	*endA1 hsdR17 supE44 thi-1 recA1 gyrA96 relA1 (argF-lacZYA)U169 80dlacZ*	[31]
*PAO1*	reference strain; genome completely sequenced	[32]
*PAO1*Δ*ampG*	*PAO1 ampG* deletion (PA4393)	[10]
*E*. *coli* 7		this work
*A*. *baumannii* 2089		this work
*V*. *cholerae* 03		this work
**Plasmids**		
pMD18-*ampG*_mut_	pMD18 vector carrying mutated *ampG* from *PAO1*	this work
pUCP24	pUC18-derived broad-host-range vector; Gm^r^	[33]
pUCP24-*ampG*_EC_	*ampG* gene from *E*. *coli* 7 cloned into pUCP24; Gm^r^	this work
pUCP24-*ampG*_AB_	*ampG* gene from *A*. *baumannii* 2089 cloned into pUCP24; Gm^r^	this work
pUCP24-*ampG*_VC_	*ampG* gene from *V*. *cholerae* 03 cloned into pUCP24; Gm^r^	this work
pUCP24-*ampG*_PA_	*ampG* gene from *PAO1* cloned into pUCP24; Gmr	this work
**pUCP24-*ampG***_**mut**_	pUCP24 vector carrying *ampG* from *PAO1* with the indicated point mutations	
pUCP24-*ampG*_PA_-A129T		this work
pUCP24-*ampG*_PA_-A129S		this work
pUCP24-*ampG*_PA_-A129P		this work
pUCP24-*ampG*_PA_-A129G		this work
pUCP24-*ampG*_PA_-A129V		this work
pUCP24-*ampG*_PA_-A129D		this work
pUCP24-*ampG*_PA_-Q131E		this work
pUCP24-*ampG*_PA_-Q131P		this work
pUCP24-*ampG*_PA_-Q131R		this work
pUCP24-*ampG*_PA_-Q131H		this work
pUCP24-*ampG*_PA_-G29R		this work
pUCP24-*ampG*_PA_-G29C		this work
pUCP24-*ampG*_PA_-G29D		this work
pUCP24-*ampG*_PA_-G29A		this work
pUCP24-*ampG*_PA_-G29V		this work
pUCP24-*ampG*_PA_-A197T		this work
pUCP24-*ampG*_PA_-A197S		this work
pUCP24-*ampG*_PA_-A197G		this work
pUCP24-*ampG*_PA_-A197D		this work

### Genetic complementation assays

The *ampG* genes of *P*. *aeruginosa* PAO1, *E*. *coli* 7, *V*. *cholerae* 03 and *A*. *baumannii* 2089 were amplified from corresponding genomic DNA templates. A *Bam*HI restriction site was added at the 5’ end of the sense primers (PA_*ampG*_-F, EC_*ampG*_-F, AB_*ampG*_-F and VC_*ampG*_-F) and a *Hind*III (or *Sal*I) restriction site was added at the 5’ end of the anti-sense primer (PA_*ampG*_-R, EC_*ampG*_-R, AB_*ampG*_-R and VC_*ampG*_-R) (Table 2). The *ampG* polymerase chain reaction (PCR) products were first cloned into the pMD18-T vector (TaKaRa, Dalian, China). The recombinant plasmid (pMD18-*ampG*) was identified initially by PCR and was then verified by sequencing. The verified pMD18-*ampG* plasmid was digested with the restriction enzymes *Bam*HI and *Hind*III (or *Sal*I). The *ampG* fragment was recovered and then ligated into the pUCP24 vector digested with the same restriction enzymes (*Bam*HI and *Hind*III/*Sal*I). The recombinant plasmid pUCP24-*ampG* was transformed into *E*. *coli* JM109, and the recombinant was further identified and verified by PCR. The plasmid pUCP24-*ampG* was extracted and introduced into *PAO1*Δ*ampG* as described previously [10]. *PAO1*Δ*ampG* carrying vector pUCP24 was used as a negative control.

**Table 2:** Primers used in this study.

Primer	Sequence^a^^,^ ^b^	Purpose
PA_*ampG*_-F	5’GGGATCCCAACGCGCACGCTTGCGCGAGGA 3’(BamHI)	Cloning of *ampG* of *PAO1*
PA_*ampG*_-R	5’GAAGCTTTCAGTGCTGCTCGGCGTTCTGGT3’(HindIII)	
EC_*ampG*_-F	5’GGGATCCATGTCCAGTCAATATTTACG 3’(BamHI)	Cloning of *ampG* of *E*.*coli* 7
EC_*ampG*_-R	5’GAAGCTTTTACGTCAGATGCGTTTTTCG 3’(HindIII)	
AB_*ampG*_-F	5’GGGATCCATGTTCCGACAGCAAAATCTTTA 3’(BamHI)	Cloning of *ampG* of *A*. *baumannii* 2089
AB_*ampG*_-R	5’GGTCGACTTATACAGTTTTAGCATCTTTCCA 3(SalI)	
VC_*ampG*_-F	5’GGGATCCTAGGTACAAGTAGTTGGGGCCAGG 3’(BamHI)	Cloning of *ampG* of *V*. *cholerae* 03
VC_*ampG*_-R	5’GGTCGACCAGTCCTATTTTAGTAGGTCATTTT 3’(HindIII)	
PA_*ampG*_-G29R	5’AACACCAGCATGGTCGGCAGgcgGGCGGCGAAGCCGAGCA 3’	G29R
PA_*ampG*_-G29C	5’AACACCAGCATGGTCGGCAGgcaGGCGGCGAAGCCGAGCA3’	G29C
PA_*ampG*_-G29D	5’AACACCAGCATGGTCGGCAGgtcGGCGGCGAAGCCGAGCA3’	G29N
PA_*ampG*_-G29A	5’AACACCAGCATGGTCGGCAGggcGGCGGCGAAGCCGAGCA3’	G29A
PA_*ampG*_-G29V	5’AACACCAGCATGGTCGGCAGgacGGCGGCGAAGCCGAGCA3’	G29V
PA_*ampG*_-29-R	5’CTGCCGACCATGCTGGTGTTCAACACCCTGTCGGTGTGGC3’	
PA_*ampG*_-A129T	5’TCGATCGCGATGTCCTGGGTggtGGAGGCGAACGCCACCA3’	A129T
PA_*ampG*_-A129S	5’TCGATCGCGATGTCCTGGGTggaGGAGGCGAACGCCACCA3’	A129S
PA_*ampG*_-A129P	5’TCGATCGCGATGTCCTGGGTgggGGAGGCGAACGCCACCA3’	A129P
PA_*ampG*_-A129G	5’TCGATCGCGATGTCCTGGGTgccGGAGGCGAACGCCACCA3’	A129G
PA_*ampG*_-A129V	5’TCGATCGCGATGTCCTGGGTgatGGAGGCGAACGCCACCA3’	A129G
PA_*ampG*_-A129D	5’TCGATCGCGATGTCCTGGGTgtcGGAGGCGAACGCCACCA3’	A129N
PA_*ampG*_-129-R	5’ACCCAGGACATCGCGATCGACGCCTACCGCCTGGAAATCG3’	
PA_*ampG*_-Q131E	5’TAGGCGTCGATCGCGATGTCctcGGTGGCGGAGGCGAACG3’	Q131E
PA_*ampG*_-Q131P	5’TAGGCGTCGATCGCGATGTCcggGGTGGCGGAGGCGAACG3’	Q131P
PA_*ampG*_-Q131R	5’TAGGCGTCGATCGCGATGTCccgGGTGGCGGAGGCGAACG3’	Q131R
PA_*ampG*_-Q131H	5’TAGGCGTCGATCGCGATGTCatgGGTGGCGGAGGCGAACG3’	Q131H
PA_*ampG*_-131-R	5’GACATCGCGATCGACGCCTACCGCCTGGAAATCGCCGAGG3’	
PA_*ampG*_-A197T	5’AGCCCAGGCAGGATCAGCAGggtGAACAACGCATAGGTCA3’	A197T
PA_*ampG*_-A197S	5’AGCCCAGGCAGGATCAGCAGggaGAACAACGCATAGGTCA3’	A197S
PA_*ampG*_-A197G	5’AGCCCAGGCAGGATCAGCAGgccGAACAACGCATAGGTCA3’	A197G
PA_*ampG*_-A197D	5’AGCCCAGGCAGGATCAGCAGgtcGAACAACGCATAGGTCA3’	A197N
PA_*ampG*_-197-R	5’CTGCTGATCCTGCCTGGGCTGGTCACCAGCCTGCTGATCC3’	
PA_*ampG*_-F_*qPCR*_	5’GCGGTCTCGGTGATGGTGCT 3’	qPCR
PA_*ampG*_-R_*qPCR*_	5’CGCTGGACGAACTCGGTGAT 3’	
EC-F_*qPCR*_	5’TGATGGACCGCTACACGC 3’	qPCR
EC-R_*qPCR*_	5’ACCCAGAACGCTGATTGC 3’	
AB-F_*qPCR*_	5’ACAGGCGCAACTCAAGAT 3’	qPCR
AB-R_*qPCR*_	5’CCCAATAAAGCAGCAACA 3’	
VC-F_*qPCR*_	5’TGACTGGCTGGCTGAAAG 3’	qPCR
VC-R_*qPCR*_	5’CGATGGATTGGCAGAAAA 3’	
RpoD-F_*qPCR*_	5’CCGTTGCTGAATATCCGGAA 3’	qPCR
RpoD-R_*qPCR*_	5’CAAATTTTTCGCGAGCCAGT 3’	

^a:^ The underlined sequences are restriction endonuclease sites.

^b:^ The nucleotide sequence corresponding to the mutated amino acids are in lowercase.

### Site-directed mutagenesis of the conserved amino acids in ampG gene

The mutant nucleotide was introduced into the 3’ end primer of the 5’ end fragment as well as the 5’ end primer of the 3’ end fragment where a 10 bp overlap was located. In total, 19 pairs of primers were designed using the online software Premier 5.0 for the four conserved amino acids from the *ampG* gene for site-directed mutagenesis (Table 2). The common sense primer (PA_*ampG*_-F) was flanked by the *Bam*HI restriction site, and the common anti-sense primer (PA_*ampG*_-R) was flanked by the *Hind*III restriction site (Table 2). For the recombinant PCR of each mutated site, a pair of the common primers was used with the corresponding 5’ and 3’ end mutated fragments as the templates. The PCRs were performed under the following conditions: an initial 5 min denaturation at 95°C, followed by 35 amplification cycles consisting of a 45 sec denaturation step at 95°C, a 55 sec annealing step at 55°C and a 45 sec extension step at 72°C, followed by a 10 min final extension at 72°C (ExTaq, TaKaRa, Dalian, China). The PCR products (*ampG*_mut_) were purified and inserted into pMD18-T vectors. The recombinant pMD18-*ampG*_mut_ plasmids were initially identified by PCR and were then verified by sequencing. The verified pMD18-*ampG*_mut_ recombinant plasmids were digested with *Bam*HI and *Hind*III. The *ampG*_mut_ fragments were recovered and ligated into pUCP24 digested with the same restriction enzymes (*Bam*HI and *Hind*III). The recombinant pUCP24-*ampG*_mut_ plasmids were then transformed into *PAO1*Δ*ampG* for the complementation studies.

### Antibiotic susceptibility testing

The minimal inhibitory concentration (MIC) of the antimicrobial agents was determined by the agar dilution method for the control and recombinant strains in accordance with the guidelines of the Clinical and Laboratory Standards Institute (CLSI). The antimicrobial agents were obtained from the National Institute for the Control of Pharmaceutical and Biological Products (NICPBP) and pharmaceutical companies in China. *E*. *coli* ATCC 25922 was used as a quality control for the MIC tests.

### Detection of β-lactamase activity

The β-lactamase detection procedures were performed as described by Zhang Y et al. [10]. *P*. *aeruginosa* cells were induced for 1 h with 4 μg/ml cefoxitin (Calbiochem, San Diego, USA) and for 2 h with 50 μg/ml cefoxitin. Crude cell extracts were prepared by sonication, and the protein content of the crude extracts was determined by using the BCA Protein Assay Reagent (Pierce, USA) and bovine serum albumin as the standard [12]. The β-lactamase activity was quantified with an UV spectrophotometer using 100 μM of nitrocefin as the substrate [10]. The activity of the β-lactamase was defined as the nanomoles of nitrocefin hydrolyzed at 30°C per min by one milligram of the protein. All the induction experiments were performed in triplicate and the results represent an average of three experiments.

### qRT-PCR analysis of ampG mRNA concentration in *ampG* mutants

To validate the transcription level of the mutant *ampGs in vivo*, qRT-PCR was performed using the StepOne^™^ RT-PCR System (Applied Biosystems, USA) [13]. Total RNAs were extracted from the control and recombinants cultured with ampicillin (100 μg/ml) using the Rneasy Plant Mini Kit (Qiagen, Germany) and were then treated with Dnase I using the Rnase-Free Dnase Set (Qiagen, Germany). cDNA was obtained by reverse transcription using the PrimeScript RT-PCR Kit (TaKaRa, Dalian). After qPCR analysis, relative quantification of the targets in each sample were calculated using rpoD as the internal control [14].

### Sequence comparison and phylogenetic analysis of *ampG*

The *ampG* sequences from different bacterial species were compared by BlastX from NCBI, and the open reading frames of the *ampG* genes were identified by MEGA5.05 to analyze the similarities of the *ampG* nucleotide sequences and the AmpG amino acid sequences. Phylogenetic trees were constructed using the maximum likelihood method, and the resulting trees were tested with bootstrap values of 100 replicates using PhyML3.0 [15].

### Prediction of the AmpG secondary structure

The AmpG secondary structure was predicted using a program at http://www.predictprotein.org/, and a Kyte-Doolittle algorithm from Lasergene 7 (DNASTAR, Madison, WI) [9] was used to predict AmpG transmembrane (TM) helices. The parameters of TOPPRED-transmembrane topology prediction were as follows: Full window size: 21, Core window size: 11, Wedge window size: 5, Using hydrophobicity file: GES-scale, Cutoff for certain transmembrane segments: 1.00, Cutoff for putative transmembrane segments: 0.60, Critical distance between 2 transmembrane segments: 2, Critical loop length: 60.

### AmpG tertiary structure modeling

The folding templates were generated using BLAST Search (DS Server) from Discovery Studio [16] and FFAS03 (http://ffas.burnham.org/ffas-cgi/cgi/document.pl) [17]. The structure model of AmpG was further established using I-TASSER according to homology modeling (http://zhanglab.ccmb.med.umich.edu/I-TASSER/) [18]. Five model structures, including 1b3u (PROTEIN PHOSPHATASE PP2A), 3o7q (L-fucose-proton symporter), 1pw4 (Glycerol-3-phosphate transporter), 2bku and 4aps (GTP-BINDING NUCLEAR PROTEIN RAN) were obtained. DS Ramachandran plot and PDBsum (www.ebi.ac.uk/thornton-srv/databases/pdbsum.Generate.html) were applied for final model evaluation [19].

## Results

### Diversity of the ampG gene

**Fig 1:**
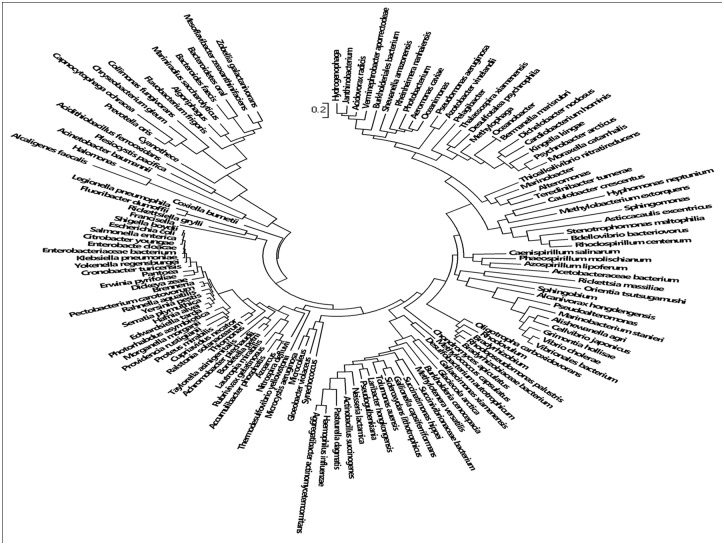
Phylogenetic tree of the *ampG* gene. The AmpG sequences from 134 species were used to generate the tree.

A total of 2245 AmpG protein related sequences were collected by searching the NCBI Protein database using AmpG as the key word. The AmpG proteins were widely distributed over different bacterial species, including 134 known genera (Fig 1, S1 Table). Using the *P*. *aeruginosa* PAO1 AmpG as a reference, the amino acid sequence similarity analysis showed that AmpG of *Azotobacter vinelandii* DJ had the highest identity of 61.9% while that of *Capnocytophaga* showed the lowest similarity of 22.7% (S1 Table). The similarities of the three AmpGs used for the genetic complementation analyses to *P*. *aeruginosa* were 53.3% (*E*. *coli* 7), 36.0% (*A*. *baumannii* 2089) and 33.0% (*V*. *cholerae* 03).

### The TM topology of AmpG

**Fig 2:**
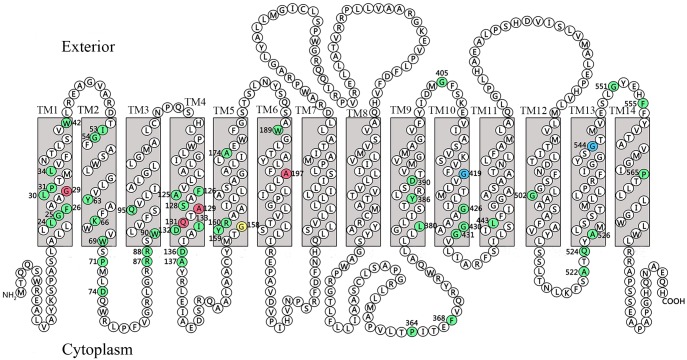
Secondary structure of AmpG from *PAO1*. The conserved amino acids are shown in green. Amino acids 29G, 129A, 131Q and 197A that are shown in red are conserved amino acids that were subjected to site-directed mutagenesis. Amino acids 419G (in blue), 544G (in blue) and 158G (in yellow) correspond to C151, G268 and G373 in AmpG of *E*. *coli* SN0301, respectively.

The length and number of TM helices in the various AmpG proteins differ significantly. The longest AmpG is from *P*. *aeruginosa* (CDR92618) [9], which consists of 598 amino acids (AA) and 14 predicted TM helices (Fig 2). The shortest AmpG is from *Microcoleus*, which only has 401 AA and 11 TM helices (S1 Table). Most AmpG proteins contain 12 or 14 TM helices. The four AmpGs used for genetic complementary analysis have 14 (*PAO1*, *E*. *coli* 7), 11 (*A*. *baumannii* 2089) and 10 TM helices (*V*. *cholerae* 03) (S1 Table).

### Conservation of AmpG

A total of 134 AmpGs from 134 genera were chosen as the representatives for conservation analysis. Using AmpG from *PAO1* as a reference, the amino acids that appeared in more than 80% of the 134 representative AmpGs were considered as conserved [9]. Excluding TM 7 and 8 in *P*. *aeruginosa* (TM 7 and 8 do not exist in many AmpGs), 10 or more TM helices were identified in nearly half (66/134) AmpG proteins (S2 Table). The results of the conserved amino acid analysis showed that there were 51 conserved amino acids all clustered in the 12 TM helices. In *P*. *aeruginosa* PAO1, these 12 conserved amino acid clusters (Table 3) were distributed over 12 of the 14 TM helices with TM 7 and 8 as the exceptions. Seven of the 12 clusters locate to TMs 1, 2, 3, 4, 9, 10 and 13. These conserved amino acid residues could be critical for maintaining the function of AmpG.

**Table 3:** The 12 conserved amino acid clusters.

	Sequence	TM
Class 1	L^24^G^25^F^26^AAG^29^L^30^P^31^TML^34^VFNTLSVW^42^	Segment no.1
Class 2	I^53^G^54^FASWLGLVY^63^AFK^66^WVW^69^SP^71^MLD^74^	Segment no.2
Class 3	R^87^R^88^SW^90^LVFSQ^95^	Segment no.3
Class 4	A^125^F^126^AS^128^A^129^TQ^131^D^132^I^133^AID^136^A^137^	Segment no.4
Class 5	Y^159^R^160^AAILLASAGALILA^174^	Segment no.5
Class 6	W^189^GLTYALFA^197^	Segment no.6
Class 7	P^364^ITEF^368^VQRYRWQALLLL^380^GLISTY^386^RLSD^390^	Segment no.9
Class 8	G^405^FSKEVIASVSKVFG^419^VLMTLIG^426^AAAG^430^G^431^	Segment no.10
Class 9	SIL^443^FIGGAAS	Segment no.11
Class 10	DNFSGG^502^LAASAFVA	Segment no.12
Class 11	A^522^TQ^524^YA^526^MLSSTMLLLPRFIGGYSG^544^TMVESLG^551^	Segment no.13
Class 12	F^555^FYVTAVMGIP^565^	Segment no.14

Note: The residues with the superscripted numbers are the conserved amino acids.

### Functional analysis of AmpG proteins cloned from different genera of bacteria

In our previous work, we used homologous recombination technology to obtain a *ampG* knockout in *P*. *aeruginosa* strain PAO1 (*PAO1*Δ*ampG*) [10]. The MIC for ampicillin decreased from 512 μg/ml (*PAO1*) to 32 μg/ml (*PAO1*Δ*ampG*) [10]. This result indicated that the *ampG* gene is essential for the induction of the AmpC type β-lactamase. Although, the *ampG* genes in different bacteria differ from each other in length and number of transmembrane regions, they share many conserved domains in the transmembrane regions. To detect the activity of various AmpGs from different origins, we performed genetic complementation analysis of *ampG*s cloned from *E*. *coli* 7 (491 AA, 14 TM helices), *V*. *cholerae* 03 (462 AA, 10 TM helices), *A*. *baumannii* 2089 (415 AA, 11 TM helices) as well as *ampG* of *P*. *aeruginosa* PAO1 (594 AA, 14 TM helices). The plasmid pUCP24 was used as the *ampG* carrier vector, and *PAO1*Δ*ampG* was the host strain. The results showed that all of the cloned *ampG* genes complemented the function of the deleted *ampG* gene, and the MIC for ampicillin was almost fully restored to the level of the *P*. *aeruginosa* PAO1 parental strain. The β-lactamase activity of the genetically complemented recombinants also showed similar results (Table 4). Overall, these results show that three genes from different bacterial species have similar functions and can compensate the function of the deleted *ampG* gene in *PAO1*Δ*ampG*, regardless of their structural differences. However, the AmpGs from *E*. *coli* and *A*. *baumannii* only showed approximately one third AmpC induction compared to *P*. *aeruginosa* or *V*. *cholerae* AmpG. The reason for this difference is unknown.

**Table 4:** Summary of ampicillin MIC, AmpC β-lactamase activity and qRT-PCR of *ampG* RNA results.

Strain	MIC(μg/ml)	β-Lactamase activity^a^		qPCR(2^-ΔΔCt^)
		Non-induced	Induced	
*ATCC 25922*	4	0	0	-
*PAO1*	512	112.40±7.2	1981.64±56	1.00±0.0
*PAO1ΔampG*	32	35.49±2.5	44.82±3.3	0.08±0.0
*PAO1ΔampG-ampG*_*PA*_	1024	37.87±1.8	5019.31±98.2	8835.75±45.4
*PAO1ΔampG-ampG*_*EC*_	512	118.50±7.0	1622.63±34	-
*PAO1ΔampG-ampG*_*AB*_	1024	49.48±4.2	1680.35±28	-
*PAO1ΔampG-ampG*_*VC*_	512	24.40±1.5	5063.64±67.3	-
*PAO1ΔampG*-UCP24-*ampG*_PA_-A129T	64	309.64±10.6	139.37±6.6	10363.27±128.3
*PAO1ΔampG*-pUCP24-*ampG*_PA_-A129S	1024	205.27±7.8	9556.45±132	6758.90±47.2
*PAO1ΔampG*-UCP24-*ampG*_PA_-A129P	1024	895.45±66.8	12450.43±524	8803.84±56.9
*PAO1ΔampG*-pUCP24-*ampG*_PA_-A129G	512	262.83±8.9	5342.68±88.7	9738.85±123.3
*PAO1ΔampG*-pUCP24-*ampG*_PA_-A129V	64	200.74±14	232.28±26	0.52±0.0
*PAO1ΔampG*-pUCP24-*ampG*_PA_-A129D	64	233.23±12.5	576.69±35	6218.63±45.6
*PAO1ΔampG*-pUCP24-*ampG*_PA_-Q131E	1024	282.83±10	12559.25±213	6097.70±78.5
*PAO1ΔampG*-pUCP24-*ampG*_PA_-Q131P	1024	101.28±5.8	12518.16±432.8	6433.10±104.6
*PAO1ΔampG*-pUCP24-*ampG*_PA_-Q131R	1024	186.91±3	12393.96±541	9838.86±218.9
*PAO1ΔampG*-pUCP24-*ampG*_PA_-Q131H	1024	187.31±2.1	11109.25±426	13156.64±321.2
*PAO1ΔampG*- pUCP24-*ampG*_PA_-G29R	512	179.91±3.8	5142.63±132	4140.62±34.0
*PAO1ΔampG*- pUCP24-*ampG*_PA_-G29C	512	117.52±4.6	4928.87±265.4	4054.96±62.6
*PAO1ΔampG*- pUCP24-*ampG*_PA_-G29D	1024	223.10±1.6	4750.43±192	9054.04±102.7
*PAO1ΔampG*- pUCP24-*ampG*_PA_-G29A	64	40.15±4	236.33±12.3	0.74±0.0
*PAO1ΔampG*- pUCP24-*ampG*_PA_-G29V	64	17.30±1	209.16±36	0.32±0.0
*PAO1ΔampG*-pUCP24-*ampG*_PA_-A197T	1024	163.03±21	3453.21±43.2	5123.37±36.9
*PAO1ΔampG*-pUCP24-*ampG*_PA_-A197S	64	32.21±2.2	145.28±5.7	0.63±0.0
*PAO1ΔampG*-pUCP24-*ampG*_PA_-A197G	1024	170.69±6	5023.37±246	7865.3±44.8
*PAO1ΔampG*-pUCP24-*ampG*_PA_-A197D	128	46.28±7.8	406.78±11.2	0.12±0.0

^a:^ Nanomoles of nitrocefin hydrolyzed per minute per milligram of protein.

^_^, not tested.

### Tertiary structure prediction of AmpG of *P*. *aeruginosa*

**Fig 3:**
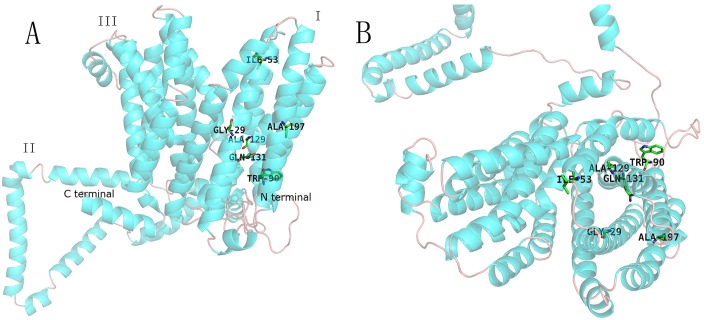
The modeled 3D structures of AmpG from *PAO1*. Twelve TM helices and one internal membrane region are illustrated. The first and third parts consist of six transmembrane α helices. The region between the first and third parts (named the second part) is a sequence of 167 AA in length. The mutated amino acids at positions 29, 53, 90, 129, 131 and 197 are illustrated in the third part (A). The picture on the right illustrates the protein as viewed from outside of the membrane (B).

**Fig 4:**
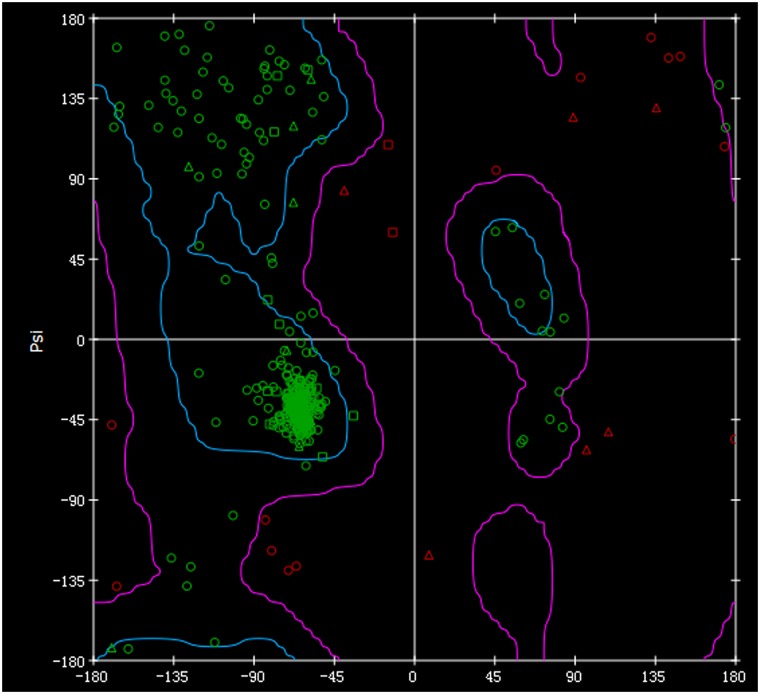
Ramachandran plots for the modeled 3D structures of AmpG from *PAO1*. The Ramachandran plot indicates low energy conformations for φ (phi) and ψ (psi). The conventional terms are used to represent the torsion angles on either side of the alpha carbons in the peptides. A triangle indicates glycine, a square indicates proline and all other types of amino acids are indicated as circles. The regions circled with gray lines represent the most favorable combinations of phi-psi values.

As the crystal structure of AmpG has not yet been resolved, we used DS Blast, FFAS03 and I-TASSER to predict the structure of AmpG from *P*. *aeruginosa* PAO1. The folding templates 1b3u, 3o7q, 1pw4, 2bku and 4aps were derived from human PROTEIN PHOSPHATASE PP2A, L-fucose-proton symporter of *E*. *coli* K-12, Glycerol-3-phosphate transporter of *E*. *coli* and GTP-BINDING NUCLEAR PROTEIN RAN of *Saccharomyces cerevisiae*, respectively. Using combined I-TASSER with two functional modules of Align Sequence to Templates and DS's Build Homology Models, 3D models of AmpG protein were created. According to the matching degree of secondary structure, the optimal structure was then chosen. The predicted 3D structure of *P*. *aeruginosa* PAO1 AmpG mainly consists of three parts (Fig 3). The first and third parts each consist of 6 transmembrane α helices that form an opening inside of the cell. The second part located between these two parts consist of 167 AA forming α helices and loop structures outside of the membrane. Both the N-terminus and C-terminus are located close to each other in the cytoplasm. Geometric evaluations of the modeled 3D structures of *P*. *aeruginosa* AmpG were performed using Discovery Studio by calculating the Ramachandran plot. The plot showed that 90.2% of residues were found in the favored regions, 8.8% were in the allowed regions and 1.0% were in the outlier region (Fig 4).

### Effect of conserved amino acid mutations on the function of AmpG

Based on the results shown above, 51 amino acids distributed in 12 clusters are conserved. To analyze the correlation of these conserved amino acids with the function of AmpG, 4 conserved amino acids (G29, A129, Q131 and A197) from the transmembrane regions were chosen for mutational analysis. These four amino acid residues are located in TM 1 (G29), 4 (A129, Q131) and 6 (A197) assembly of the first part of the protein (Fig 2). According to the chemical properties of the amino acids, 4 to 6 different amino acid substitutions were designed for each of the conserved amino acids. A total of 19 mutated *ampG* genes were generated (Table 1), and these gene clones were transformed into recipient *PAO1*Δ*ampG* cells. The MICs for ampicillin and the β-lactamase activities were detected (Table 4). The transcription levels of the *ampG* mutants were examined by qRT-PCR. As the results show, most mutants conferred strong resistance to ampicillin, similar to the original *P*. *aeruginosa* PAO1 strain. The G29A, G29V, A129T, A129V, A129D, A197S, and A197D mutants conferred significantly lower resistance to ampicillin and had significantly lower β-lactamase activities (Table 4).

Similar to *PAO1*Δ*ampG*, the expression level of *ampG* containing the G29A, G29V, A129V, A197S and A197D mutations decreased significantly (Table 4), suggesting that amino acids at position 29, 129 and 197 have strong effects on the expression of *ampG*. Conversely, the expression levels of the A129T and A129D mutants were close to *PAO1*Δ*ampG*-*ampG*_PAO1_ and the rest of the mutants. However, the MICs for ampicillin and the β-lactamase activities of the A129T and A129D mutants were similar to *PAO1*Δ*ampG*, suggesting that the mutants with A129T and A129D did not affect *ampG* expression but rather AmpG function was drastically decreased.

## Discussion

The gene *ampG* is encoded on the chromosome and is widely distributed in many genera of bacteria. AmpG is a transmembrane protein that acts as a permease and transports muropeptide from the periplasm into the cytoplasm. The muropeptide derivatives are essential for the induction of AmpC type β-lactamase expression [20]. It was previously reported that the main material transported by AmpG is GlcNAc-anhMurNAc, and muropeptides that lack GlcNAc or anhMurNAc are not transported [21]. Dietz D et al. indicated that AmpG is responsible for the transportation of N-acetylglucosamine-1,6-anhydro-N-acetylmuramic acid- tetrapeptide aldehyde from the periplasm into the cytoplasm, which is generated from the degradation of murein by hydrolases [22]. The degraded products of murein are transferred to AmpD by AmpE and are then hydrolyzed by AmpD or are first degraded by β-N-acetyl-glucosaminidase to N-acetyl-muramyl tripeptide in the cytoplasm and then hydrolyzed by AmpD. When N-acetyl-muramyl tripeptide binds with AmpR, it may activate AmpR and induce transcription of *ampC* [23]. Deletion or mutation of *ampG* may cause the cell membrane to lack AmpG completely or may result in a structural abnormality in AmpG. Lack of or presence of a mutant form of AmpG may result in a defect in transport of N-acetyl-muramyl tripeptide into the cytoplasm, thereby reducing AmpC type β-lactamase induction [24–26].

Although AmpG was found to be widely distributed throughout gram-negative bacteria, it does vary in size and in the number of TM helices. However, secondary structure prediction showed that a large part of the TM helices are conserved in most bacteria. The AmpG protein in one type of *E*. *coli* contained 14 TM helices, and there were four hydrophobic segments within the cytoplasm. In this work, AmpG proteins from different genera of the bacteria (*P*. *aeruginosa* PAO1, 14, *V*. *cholerae* 03, 10, *A*. *baumannii* 2089, 11 and *E*. *coli* 7, 14) were used to perform genetic complementation experiments. The AmpG proteins in these bacteria have different numbers of TM helices. The results presented here showed that all of the AmpG proteins could function properly in the recipient *PAO1*Δ*ampG*. Similar to the wild type, the *ampG* complemented *PAO1*Δ*ampG* strains regained high level ampicillin MICs and expressed AmpC type β-lactamase with normal activity. Similar experiments have been performed by Huang et al., who showed that the AmpG of *E*. *coli* can replace a mutated *ampG* or *ampN* in *Stenotrophomonas maltophilia* and restore β-lactamase production [27]. In this work, although the donor AmpG proteins had different numbers of TM helices, and similarity between the TM helices of four AmpGs were only 33–53%, they all contain 10 (*V*. *cholerae* 03), 11 (*baumannii* 2089) or 14 (*E*. *coli* 7 and *P*. *aeruginosa* PAO1) clusters of conserved amino acid residues. Schmidt et al. [24] used nitrosoguanidine (NTG) to induce mutations in *E*. *coli* SN0301 (carrying *ampC* and *ampR* of *E*. *cloacae*) and obtained three non-functional *ampG* gene mutants, including *ampG1* (G151-D151), *ampG3* (G268-D268) and *ampG5* (G373-D373). The three mutated amino acids correspond to positions 158 (G), 419 (G) and 544 (G) in AmpG of *P*. *aeruginosa* PAO1, and each amino acid position is in the conserved amino acid regions located in TM 5, 10 and 13 in AmpG of *P*. *aeruginosa* PAO1, respectively. Mutation of *ampG* can lead to the functional loss of the AmpG protein, resulting in a failure to induce β-lactamase or a significant reduction in AmpC type of β-lactamase induction. It is suggested that the conserved amino acids play an important role in maintaining normal AmpG function.

A single amino acid might be enough to determine the substrate specificity and transportation activity of AmpG. The expression levels of *ampG* mRNA in the G29A, G29V, A129V, A197S and A197D mutants were significantly decreased in PAO1Δ*ampG* compared to the wild type *ampG*. Conversely, when A129 was mutated to D or T, the transcriptional levels of *ampG* was unchanged, but the ampicillin MIC levels and AmpC type β-lactamase activities were drastically reduced. These results indicate that although the mutations did not affect the transcription of *ampG*, the AmpG structure might have changed and resulted in a loss of function. Structural and biochemical tests of the protein LacY showed that co-transport of protons involve a complex network of salt bridges and hydrogen bonds (H-bond) [28]. Some amino acid residues are essential for the protein functions of binding, transferring and releasing of protons. The function of glutamate at position 269 is to bind with a proton and the lactose substrate and subsequently induces proton transfer to the glutamic acid at position 325. This process drives a conformational change of the protein into an inward opening. The substrate is released into the cell, then the proton is released from the glutamic acid at position 325, the protein then changes conformation to the outward opening position, and another cycle begins [28, 29]. In this study, it was predicted that the N- and C-terminus of *P*. *aeruginosa* PAO1 AmpG are located in the cytoplasm and that they are spatially close to each other. The binding site for the substrate is in the center of the cavity located in the outer end of the transmembrane channel formed by the transmembrane regions. When the substrate binds to the cavity, it will lead to a conformational change of the protein so that the inner end of the channel opens and drives the entrance of the substrate into the cytoplasm [30]. The amino acids utilized in this work (G29, A129, A197) were all conserved and were located in the first part of the transmembrane regions. Mutation of these residues might cause a steric effect and/or electrostatic force that prevents the transmembrane protein from binding to the substrate or hinders the conformational change of AmpG when it binds to the substrate. Thus, the normal function of AmpG is diminished. When the muropeptides (such as N-acetylglucosamine-1,6-anhydro-N-acetylmuramic acid-tetrapeptide aldehyde), which are the initial substrate of the inducer for the AmpC type β-lactamase, cannot be transported by AmpG to cytoplasm, the AmpC type β-lactamase level in the cell would be diminished as a consequence.

With the rapid emergence of antibiotic resistance, curing infectious diseases is a serious challenge in the clinical setting. It is critically important to study the regulatory mechanism of the expression of AmpC type β-lactamase at a cellular level. Agents that interfere with the function of AmpG may hinder the transportation of the muropeptide and decrease the level of AmpC type β-lactamase in the cell, which in turn increases the sensitivity of the cell to commonly used anti-bacterial agents. This study suggests a new research avenue: utilize AmpG as a drug target and conduct rational design and inhibitor screens with the goal of overcoming bacterial resistance to β-lactam antibiotics for clinical therapy of infectious diseases.

## Supporting Information

S1 Table Distribution of 2245 AmpGs over 134 genera of bacteria. (DOC)

S2 Table The result of conservative amino acid cluster analysis. (DOC)
