# Supplementary material for: Structure-Function Analysis of the Transmembrane Protein AmpG from Pseudomonas aeruginosa
Source: PLoS One. 2016 Dec 13;11(12):e0168060. doi: 10.1371/journal.pone.0168060 (PMC5154545; doi:10.1371/journal.pone.0168060)
Supplement: S1 Table — (DOC) [file pone.0168060.s001.doc]

Table S1 Distribution of 2245 AmpGs over 134 genera of bacteria

| Genus | Number | Percentage | Max length | Min length | Reference | Max identity* | Length | Transmembrane domain |
| --- | --- | --- | --- | --- | --- | --- | --- | --- |
|  |  |  | (aa) | (aa) |  |  | (aa) |  |
| Escherichia | 676 | 0.3011 | 497 | 426 | gi|209743901 | 53 | 491 | 14 |
| Rickettsia | 202 | 0.0900 | 572 | 408 | gi|402703676 | 38.28 | 414 | 12 |
| Pseudomonas | 198 | 0.0882 | 598 | 405 | gi|110645304 | 100 | 594 | 14 |
| Yersinia | 145 | 0.0646 | 510 | 422 | gi|170022262 | 38 | 492 | 14 |
| Vibrio | 128 | 0.0570 | 482 | 420 | gi|229606122 | 33 | 462 | 10 |
| Neisseria | 112 | 0.0499 | 523 | 408 | gi|327460779 | 47.69 | 522 | 13 |
| Prevotella | 76 | 0.0339 | 461 | 401 | gi|288929046 | 32.47 | 422 | 12 |
| Klebsiella | 44 | 0.0196 | 491 | 491 | gi|386032579 | 43 | 491 | 14 |
| Shigella | 36 | 0.0160 | 491 | 453 | gi|82542618 | 53 | 491 | 14 |
| Salmonella | 36 | 0.0160 | 505 | 402 | gi|379699217 | 38 | 491 | 14 |
| Bacteroides | 27 | 0.0120 | 452 | 416 | gi|298260409 | 23.4 | 435 | 12 |
| Aggregatibacter | 26 | 0.0116 | 432 | 413 | gi|251792233 | 30 | 424 | 12 |
| Moraxella | 24 | 0.0107 | 540 | 514 | gi|407812967 | 39 | 540 | 14 |
| Microcystis | 24 | 0.0107 | 445 | 413 | gi|389731866 | 43.9 | 413 | 12 |
| Burkholderia | 24 | 0.0107 | 473 | 428 | gi|83654242 | 35 | 437 | 10 |
| Haemophilus | 23 | 0.0102 | 428 | 412 | gi|386263233 | 33 | 425 | 12 |
| Pseudoalteromonas | 18 | 0.0080 | 459 | 458 | gi|358027620 | 44 | 458 | 12 |
| Enterobacter | 16 | 0.0071 | 499 | 491 | gi|296100371 | 43 | 491 | 14 |
| Cronobacter | 16 | 0.0071 | 495 | 488 | gi|426310398 | 31.34 | 493 | 14 |
| Pantoea | 14 | 0.0062 | 532 | 495 | gi|316948788 | 33.93 | 497 | 14 |
| Legionella | 14 | 0.0062 | 423 | 415 | gi|397663356 | 28.11 | 419 | 13 |
| Glaciecola | 14 | 0.0062 | 520 | 414 | gi|410141578 | 50 | 425 | 10 |
| Coxiella | 14 | 0.0062 | 433 | 433 | gi|165917678 | 29.39 | 433 | 10 |
| Serratia | 13 | 0.0058 | 492 | 492 | gi|440051433 | 30.74 | 492 | 14 |
| Pasteurella | 12 | 0.0053 | 428 | 425 | gi|387769856 | 28.46 | 428 | 9 |
| Francisella | 11 | 0.0049 | 457 | 403 | gi|336446142 | 33.33 | 403 | 12 |
| Bradyrhizobium | 10 | 0.0045 | 460 | 460 | gi|365901510 | 31.92 | 460 | 12 |
| Taylorella | 8 | 0.0036 | 433 | 417 | gi|347974621 | 32.13 | 433 | 12 |
| Edwardsiella | 8 | 0.0036 | 505 | 493 | gi|238919007 | 31.71 | 493 | 14 |
| Aeromonas | 8 | 0.0036 | 467 | 433 | gi|145300105 | 53 | 459 | 12 |
| Stenotrophomonas | 7 | 0.0031 | 457 | 434 | gi|293338472 | 35.06 | 457 | 12 |
| Ralstonia | 6 | 0.0027 | 460 | 429 | gi|392717698 | 33.64 | 441 | 12 |
| Pectobacterium | 6 | 0.0027 | 495 | 495 | gi|251754017 | 29.44 | 495 | 14 |
| Oligotropha | 6 | 0.0027 | 456 | 456 | gi|386028853 | 32.26 | 456 | 12 |
| Citrobacter | 6 | 0.0027 | 491 | 491 | gi|394718222 | 29.49 | 491 | 14 |
| Chondromyces | 6 | 0.0027 | 427 | 403 | gi|397086244 | 36.26 | 427 | 11 |
| Alishewanella | 6 | 0.0027 | 460 | 456 | gi|392606254 | 37.79 | 460 | 12 |
| Shewanella | 5 | 0.0022 | 466 | 439 | gi|24350114 | 52.43 | 461 | 12 |
| Proteus | 5 | 0.0022 | 506 | 431 | gi|225202259 | 32.18 | 431 | 12 |
| Thalassospira | 4 | 0.0018 | 526 | 521 | gi|407288860 | 45.99 | 526 | 14 |
| Rahnella | 4 | 0.0018 | 492 | 492 | gi|383191246 | 31.82 | 492 | 14 |
| Pseudogulbenkiania | 4 | 0.0018 | 421 | 421 | gi|347541450 | 30.63 | 421 | 11 |
| Providencia | 4 | 0.0018 | 503 | 500 | gi|261346368 | 27.6 | 503 | 14 |
| Methylophaga | 4 | 0.0018 | 541 | 516 | gi|387131111 | 47.83 | 516 | 14 |
| Methylobacterium | 4 | 0.0018 | 482 | 482 | gi|240140856 | 40.78 | 482 | 12 |
| Marinobacter | 4 | 0.0018 | 444 | 439 | gi|387814902 | 46 | 439 | 12 |
| Kingella | 4 | 0.0018 | 506 | 505 | gi|380834690 | 46.77 | 505 | 14 |
| Erwinia | 4 | 0.0018 | 495 | 493 | gi|224964880 | 31.25 | 495 | 14 |
| Dickeya | 4 | 0.0018 | 489 | 489 | gi|247539273 | 29.49 | 489 | 13 |
| Cellvibrio | 4 | 0.0018 | 478 | 431 | gi|192358898 | 35.14 | 478 | 12 |
| Capnocytophaga | 4 | 0.0018 | 427 | 422 | gi|213963247 | 22.73 | 422 | 12 |
| Bradyrhizobiaceae | 4 | 0.0018 | 455 | 406 | gi|338234624 | 32.93 | 455 | 12 |
| Azospirillum | 4 | 0.0018 | 455 | 449 | gi|356877598 | 45.36 | 449 | 12 |
| Alcaligenes | 4 | 0.0018 | 413 | 411 | gi|393163028 | 30.23 | 413 | 12 |
| Actinobacillus | 4 | 0.0018 | 440 | 428 | gi|322515289 | 31.21 | 440 | 12 |
| Achromobacter | 4 | 0.0018 | 427 | 426 | gi|293603133 | 31.69 | 427 | 12 |
| Photorhabdus | 3 | 0.0013 | 494 | 492 | gi|36787169 | 26.91 | 492 | 14 |
| Janthinobacterium | 3 | 0.0013 | 448 | 419 | gi|152982867 | 49.31 | 448 | 12 |
| Hydrogenophaga | 3 | 0.0013 | 463 | 463 | gi|388263791 | 50.55 | 463 | 11 |
| Halomonas | 3 | 0.0013 | 453 | 416 | gi|357969069 | 50.94 | 453 | 12 |
| Collimonas | 3 | 0.0013 | 433 | 418 | gi|340789331 | 33.91 | 418 | 10 |
| Caulobacter | 3 | 0.0013 | 559 | 559 | gi|220962247 | 38.19 | 559 | 11 |
| Alcanivorax | 3 | 0.0013 | 514 | 449 | gi|110833491 | 42.93 | 514 | 13 |
| Acidovorax | 3 | 0.0013 | 562 | 464 | gi|407894828 | 46 | 464 | 11 |
| Zobellia | 2 | 0.0009 | 424 | 424 | gi|339734482 | 25.81 | 424 | 12 |
| Yokenella | 2 | 0.0009 | 494 | 494 | gi|365848310 | 29.49 | 494 | 14 |
| Vibrionales | 2 | 0.0009 | 442 | 442 | gi|145961490 | 40.83 | 442 | 10 |
| Tolumonas | 2 | 0.0009 | 412 | 412 | gi|237499831 | 34.48 | 412 | 11 |
| Thiomonas | 2 | 0.0009 | 449 | 449 | gi|294341696 | 37.5 | 449 | 12 |
| Thioalkalivibrio | 2 | 0.0009 | 442 | 442 | gi|430763010 | 44.92 | 442 | 12 |
| Thermodesulfovibrio | 2 | 0.0009 | 403 | 403 | gi|206890001 | 30.65 | 403 | 12 |
| Teredinibacter | 2 | 0.0009 | 428 | 428 | gi|254786973 | 47.2 | 428 | 12 |
| Synechococcus | 2 | 0.0009 | 448 | 448 | gi|86557440 | 31.22 | 448 | 12 |
| Succinatimonas | 2 | 0.0009 | 427 | 427 | gi|322416025 | 30.88 | 427 | 10 |
| Sphingomonas | 2 | 0.0009 | 533 | 448 | gi|393725089 | 35.71 | 533 | 13 |
| Sphingobium | 2 | 0.0009 | 452 | 452 | gi|347527748 | 28.7 | 452 | 11 |
| Sideroxydans | 2 | 0.0009 | 419 | 419 | gi|291582618 | 29.84 | 419 | 10 |
| Rubrivivax | 2 | 0.0009 | 525 | 525 | gi|381381192 | 33.65 | 525 | 14 |
| Rickettsiella | 2 | 0.0009 | 415 | 415 | gi|159121137 | 28.44 | 415 | 12 |
| Rhodovulum | 2 | 0.0009 | 469 | 469 | gi|402498515 | 28.38 | 469 | 12 |
| Rheinheimera | 2 | 0.0009 | 444 | 444 | gi|383705371 | 50 | 444 | 12 |
| Psychrobacter | 2 | 0.0009 | 550 | 550 | gi|71038777 | 41.04 | 550 | 14 |
| Plesiocystis | 2 | 0.0009 | 540 | 540 | gi|149920476 | 23.86 | 540 | 12 |
| Photobacterium | 2 | 0.0009 | 460 | 460 | gi|441504634 | 50 | 460 | 12 |
| Phaeospirillum | 2 | 0.0009 | 445 | 445 | gi|381168085 | 41.28 | 445 | 10 |
| Orientia | 2 | 0.0009 | 420 | 420 | gi|189181093 | 24.59 | 420 | 12 |
| Oceanimonas | 2 | 0.0009 | 433 | 433 | gi|374335006 | 55.38 | 433 | 11 |
| Nitrospira | 2 | 0.0009 | 421 | 421 | gi|302037263 | 31.53 | 421 | 10 |
| Morganella | 2 | 0.0009 | 495 | 495 | gi|409766249 | 30.32 | 495 | 14 |
| Microcoleus | 2 | 0.0009 | 401 | 401 | gi|428253095 | 42.78 | 401 | 11 |
| Methylotenera | 2 | 0.0009 | 422 | 422 | gi|297259103 | 33.14 | 422 | 9 |
| Mariniradius | 2 | 0.0009 | 422 | 422 | gi|436483122 | 25.97 | 422 | 12 |
| Lautropia | 2 | 0.0009 | 510 | 510 | gi|319945185 | 32.04 | 510 | 14 |
| Laribacter | 2 | 0.0009 | 422 | 422 | gi|226941886 | 30.96 | 422 | 10 |
| Hyphomonas | 2 | 0.0009 | 578 | 578 | gi|114800449 | 35.42 | 578 | 14 |
| Hafnia | 2 | 0.0009 | 493 | 493 | gi|365835448 | 31.39 | 493 | 14 |
| Grimontia | 2 | 0.0009 | 464 | 464 | gi|262221322 | 40.85 | 464 | 12 |
| Gallionella | 2 | 0.0009 | 420 | 420 | gi|302877371 | 35.03 | 420 | 10 |
| Gallaecimonas | 2 | 0.0009 | 414 | 414 | gi|407202383 | 30.18 | 414 | 10 |
| Flavobacterium | 2 | 0.0009 | 425 | 425 | gi|381188370 | 25 | 425 | 12 |
| Enterobacteriaceae | 2 | 0.0009 | 491 | 491 | gi|440288756 | 29.49 | 491 | 14 |
| Desulfotalea | 2 | 0.0009 | 519 | 519 | gi|50877816 | 45.95 | 519 | 14 |
| Desulfobacterium | 2 | 0.0009 | 431 | 431 | gi|224370804 | 34.04 | 431 | 11 |
| Cyanothece | 2 | 0.0009 | 433 | 433 | gi|256589663 | 33.08 | 433 | 12 |
| Cupriavidus | 2 | 0.0009 | 419 | 419 | gi|339324363 | 32.24 | 419 | 12 |
| Cardiobacterium | 2 | 0.0009 | 544 | 544 | gi|258520335 | 48.85 | 544 | 14 |
| Caenispirillum | 2 | 0.0009 | 437 | 437 | gi|425881707 | 42.39 | 437 | 11 |
| Burkholderiales | 2 | 0.0009 | 479 | 479 | gi|302861047 | 51.72 | 479 | 12 |
| Brenneria | 2 | 0.0009 | 491 | 491 | gi|354598754 | 27.19 | 491 | 14 |
| Bacteroidetes | 2 | 0.0009 | 418 | 418 | gi|298274558 | 24.49 | 418 | 12 |
| Azotobacter | 2 | 0.0009 | 515 | 515 | gi|226718397 | 61.92 | 515 | 14 |
| Azoarcus | 2 | 0.0009 | 506 | 506 | gi|119672677 | 34.22 | 506 | 14 |
| Algoriphagus | 2 | 0.0009 | 421 | 421 | gi|311746001 | 27.27 | 421 | 12 |
| Acinetobacter | 2 | 0.0009 | 415 | 415 | gi|213155370 | 36 | 415 | 11 |
| Acidithiobacillus | 2 | 0.0009 | 412 | 412 | gi|218518715 | 25.23 | 412 | 9 |
| Acetobacteraceae | 2 | 0.0009 | 423 | 423 | gi|365856630 | 40.94 | 423 | 10 |
| Accumulibacter | 2 | 0.0009 | 519 | 519 | gi|257092315 | 34.2 | 519 | 14 |
| Xenopus | 1 | 0.0004 | 444 | 444 | gi|301630873 | 59.17 | 444 | 11 |
| Verminephrobacter | 1 | 0.0004 | 457 | 457 | gi|347821877 | 43.14 | 457 | 11 |
| Succinivibrionaceae | 1 | 0.0004 | 472 | 472 | gi|375337266 | 26.82 | 472 | 12 |
| Rhodospirillum | 1 | 0.0004 | 469 | 469 | gi|209964275 | 49.37 | 469 | 10 |
| Rhodopseudomonas | 1 | 0.0004 | 432 | 432 | gi|39647542 | 31.9 | 432 | 12 |
| Oceanobacter | 1 | 0.0004 | 541 | 541 | gi|94425774 | 41.86 | 541 | 14 |
| Methylococcus | 1 | 0.0004 | 409 | 409 | gi|53802368 | 40.66 | 409 | 12 |
| Mesoflavibacter | 1 | 0.0004 | 422 | 422 | gi|372223831 | 26.94 | 422 | 12 |
| Marinobacterium | 1 | 0.0004 | 443 | 443 | gi|372270748 | 33.33 | 443 | 10 |
| Gloeobacter | 1 | 0.0004 | 442 | 442 | gi|37521237 | 32.13 | 442 | 11 |
| Fluoribacter | 1 | 0.0004 | 420 | 420 | gi|388456915 | 27.73 | 420 | 10 |
| Dichelobacter | 1 | 0.0004 | 511 | 511 | gi|146232982 | 45.58 | 511 | 14 |
| Bordetella | 1 | 0.0004 | 428 | 428 | gi|163258452 | 31.74 | 428 | 12 |
| Bermanella | 1 | 0.0004 | 541 | 541 | gi|94502101 | 41.86 | 541 | 14 |
| Bdellovibrio | 1 | 0.0004 | 435 | 435 | gi|426402334 | 43.17 | 435 | 13 |
| Asticcacaulis | 1 | 0.0004 | 431 | 431 | gi|315418117 | 29.03 | 431 | 10 |
| Alteromonas | 1 | 0.0004 | 451 | 451 | gi|372269058 | 43.93 | 451 | 12 |

*compared to the ampG (gi 110645304) of Pseudomonas
